# Imaging risk factors for predicting postoperative complications of intramedullary nailing for tibial fracture

**DOI:** 10.1007/s00068-024-02480-4

**Published:** 2024-02-29

**Authors:** Miao He, Xiaoxing Zhang, Tianjun Cheng, Jianhua Hu, Jie Li

**Affiliations:** Department of Orthopedic Surgery, Chongqing Emergency Medical Center (Chongqing University Central Hospital), Jiankang Road 1, Chongqing, 400010 China

**Keywords:** Intramedullary nail, Tibial fracture, Complications

## Abstract

**Objective:**

The objective of this study was to analyze the ratio of fracture site diameter to tibial isthmus diameter (TFI ratio) as a predictor of postoperative complications, including malunion and delayed union, after tibial intramedullary nailing for middle and lower tibial fractures.

**Methods:**

Data were collected from all adult patients older than 20 years of age who underwent tibial intramedullary nailing for middle and lower tibial fractures between January 2015 and January 2023 and were followed up for at least 1 year. Diabetes history, smoking history, fracture type, injury mechanism, surgical method, surgical approach, diameter of the medullary cavity at the fracture site, and diameter of the tibial isthmus were recorded. Logistic regression analysis was used to determine the variables affecting the occurrence of complications. The TFI ratio was used to calculate the sensitivity and specificity of the parameters, and ROC curves were generated to establish TFI ratio thresholds for predicting complications.

**Results:**

A total of 123 patients with middle and lower tibial fractures were treated with intramedullary nails. The mean age of the patients was 43.72 years (range, 21–81 years); 89 were males, and 34 were females. Univariate logistic regression analysis showed that fracture type, open reduction surgery, superior patellar approach, and TFI ratio were significantly correlated with postoperative complications after intramedullary nailing of a tibial fracture. Multivariate logistic regression analysis showed that the TFI ratio was an independent risk factor for complications (*P* = 0.001*). By using the TFI ratio as a predictor of complications, an ROC curve was generated to establish a threshold. The ROC curve showed that a TFI ratio ≥ 1.31 had a sensitivity of 0.89, a specificity of 0.71, and an area under the ROC curve of 0.82 for predicting complications.

**Conclusions:**

The results of this study suggest that a wider intramedullary diameter and a shorter fixed length at the fracture site are associated with a higher incidence of complications after tibial intramedullary nailing. The TFI ratio may be used as a reliable parameter for predicting complications after such surgery. In patients with a high TFI ratio (≥ 1.31), additional reduction and fixation techniques may be needed to obtain and maintain fracture reduction.

## Introduction

Tibial fractures are a very common type of long bone fracture, accounting for 37% of all lower limb fractures, and tibial intramedullary nails have become the standard treatment for tibial fractures [1, 2]. Because intramedullary nails cause little damage to soft tissue and good protection to the blood supply of bone tissue, the fracture healing rate is high, and the complications are low [3]. However, due to the enlarged medullary cavity of the distal tibial isthmus and the shorter distal fracture, intramedullary nail fixation of the distal fracture has only a short effective working length and cannot achieve rigid contact with the distal cortical bone, thus reducing the stability of distal isthmus fracture fixation. Therefore, postoperative complications often occur in this situation. This can lead to long-term residual pain and functional disability, often requiring resurgery. This will result in more clinical burden and higher treatment costs as well as more suffering and poorer outcomes for patients [4].

The risk after intramedullary nailing is currently difficult to predict [5], and if we can better identify the risk factors that lead to these complications, we can better limit them in the future and reduce the complications associated with tibial fractures. In a biomechanical study, the “distal effective working length (EWLD) of long bone fractures” was shown to predict the occurrence of complications after intramedullary nailings, and fractures with shorter distal effective working lengths were particularly prone to nonunion [6]. Due to the widening of the distal medullary cavity and the lack of good cortical bone to support the intramedullary nail, fixation of long bone subisthmic fractures is more difficult [7].

**Fig. 1:**
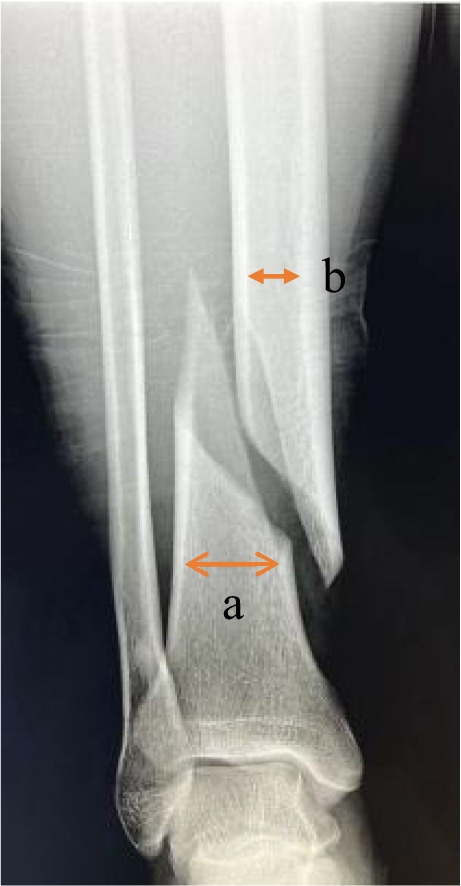
Anteroposterior radiograph. (**a**) The diameter of the fracture site was determined by the farthest intramedullary diameter of the fracture site; (**b**) diameter of the tibial isthmus; the “TFI ratio value” is defined as the ratio of the fracture site diameter to the intramedullary diameter of the tibial isthmus, which is a/b

In this study, we assessed the ratio of the tibial fracture site diameter to the isthmus diameter (TFI ratio), which was defined as the ratio of the diameter of the farthest end point of the fracture site to the diameter of the spinal canal at the isthmus (Fig. 1). This parameter was first proposed and applied to predict postoperative complications of tibial fractures with intramedullary nailing. Because the distal tibia is horn-shaped, the farther it is from the isthmus, the wider the tibial pulp cavity and the shorter the fracture distal segment. Therefore, the TFI ratio is significantly correlated with the diameter of the pulp cavity at the fracture site and the distance from the isthmus at the fracture site. The higher the ratio, the wider the pulp cavity at the fracture site and the shorter the effective working length of the intramedullary nail. The objective of this study was to analyze the reliability of the TFI as a predictor of postoperative complications, including malunion and delayed union, after intramedullary nail surgery.

## Materials and methods

This retrospective study was conducted at a single medical center and was approved by an institutional review board. Data were collected from all adult patients older than 20 years of age who underwent tibial intramedullary nailing of mid-lower tibial fractures between January 2015 and January 2023 and were followed up for at least 1 year. The inclusion criteria were as follows: (1) patients aged older than 20 years, (2) patients with old fractures or pathological fractures, (3) patients who received conservative treatment, and (4) patients who refused follow-up or were lost to follow-up. At our facility, fractures of the lower middle tibia were treated by one of five senior orthopedic surgeons who fixed them using a similar surgical approach and postoperative rehabilitation protocol. Five surgeons took turns managing orthopedic trauma patients. Diabetes history, smoking history, fracture type, injury mechanism, surgical method, surgical approach, diameter of the medullary cavity at the fracture site, and diameter of the tibial isthmus were recorded.

### Surgical techniques

#### Subpatellar approach

The knee flexion of the patient was approximately 100°. A 5–7 cm incision was made longitudinally below the anterior patellar tendon, and the patellar tendon was split longitudinally or entered from the medial patellar tendon. Regarding entry point, the lateral tubercle of the tibial intercondylar eminence (the medial edge of the lateral tibial spinous process) was coaxial with the medullary cavity, and the leading edge of the tibial plateau was inclined forward 10° to allow entry of the guide needle. After the fracture was reduced, the intramedullary nail was inserted by opening, reaming and locking the proximal and distal ends of the nail.

#### Suprapatellar approach

The patient’s knee flexion was approximately 30°. An incision of approximately 2 cm was made at the upper pole of the patella. The incision was separated from the surface of the quadriceps muscle to the joint cavity. The sleeve was inserted into the articular cavity along the intertubercular sulcus to the surface of the tibia, and the penetrating point was located on the medial margin of the lateral spinous process of the tibia in line with the lateral edge of the anterior cortex and the tibial plateau. The guide wire was inserted 10° forward, the fracture was reduced, the intramedullary nail was inserted, and the proximal and distal nails were locked.

### Postoperative follow-up

The authors reviewed all the radiographs taken at the time of injury and at 3, 6, and 12 months after surgery. Fracture union was assessed using the RUST (radiographic union scale for tibial fractures) criteria, and fracture deformity angles were recorded. RUST is used to assess the healing of the fracture by examining four layers of cortex shown on anteroposterior and lateral X-rays. The score without callus formation and visible fracture line is 1, the score with callus and visible fracture line is 2, and the score with callus and visible fracture line is 3 (Table 1) [8]. If there was no cortex score of 3, the fracture was considered to have definitely not healed, and if there was a bridging callus in 3 of the four cortices, the fracture was considered to have healed [9–11].

**Table 1:** Radiographic union scale for tibial fractures (RUST)

Score per cortex	Callus	Fracture line
1	Absent	Visible
2	Present	Visible
3	Present	Not visible in the callus bridge

### Evaluation of imaging data

Radiological images were reviewed by a radiologist and a researcher who was not involved in the surgery. Fracture union was determined by the agreement of the two raters, and all other radiological measures were taken twice by the two raters and then averaged. To determine the isthmus diameter at the time of injury, the intramedullary diameter of the middle third of the tibia was measured. This was determined by measuring the diameter of the farthest medullary cavity at the fracture site. The TFI ratio was defined as the ratio of the diameter of the fracture site to the diameter of the tibial isthmus.

Complications included delayed fracture union and malunion. Fracture union criteria were assessed by the tibial fracture imaging healing score system (RUST): at least 3 cortical articulations and bridging calluses observed on anteroposterior and lateral radiographs indicated fracture union. Malunion was defined as an angle of > 5° between the proximal medullary axis and the distal medullary axis of the tibial fracture on any plane [11]. Delayed fracture union was defined as no bone union 10 months after surgery and no improvement in the healing process for 3 consecutive months. The receiver operating characteristic (ROC) curve was used to establish the TFI ratio threshold.

### Statistical methods

All data obtained in this study were recorded and analyzed using the SPSS software package (IBM SPSS Statistics 26). Univariate logistic regression analysis was used to determine the variables affecting the occurrence of complications, and multivariate logistic regression analysis was carried out to test the independence of each variable. The TFI ratio was used to calculate the sensitivity and specificity of the parameters, and ROC curves were generated to establish TFI ratio thresholds for predicting complications.

## Results

A total of 123 patients with middle and lower tibial fractures were treated with intramedullary nails. The mean age of the patients was 43.72 years (range, 21–81 years); 89 were males, and 34 were females. There were 6 patients with diabetes, 35 patients who were smokers, 85 patients who sustained a high-energy injury, 34 patients with an open fracture, 36 patients with a transverse fracture, 49 patients with a spiral fracture, 39 patients with a comminuted fracture, 83 patients who underwent closed reduction and intramedullary nailing, and 37 patients who underwent intramedullary nailing in which the nail was inserted into the tibia via the patella. Complications occurred in 28 patients, with an incidence of 22.4% (Table 2). Twenty-two patients had malunion, and 6 patients had delayed union. During follow-up, 2 patients were found to have a postoperative infection and were excluded from the statistical data.

**Table 2:** Univariate logistic analysis of the baseline characteristics and the risk factors associated with postoperative complications

	All patients	No complications group	Complication group	OR (CI 95%)	*P*
No. of patients	123	95	28		
Sex					
Male	89(72.4%)	65(52.8%)	24(19.5%)	2.769(0.883 ~ 8.689)	0.081
Female	34(27.6%)	30(24.4%)	4(3.3%)		
Age	43.72 ± 1.35	43.05 ± 1.537	45.96 ± 2.831	1.013(0.985 ~ 1.042)	0.365
Fracture type				2.102(1.162 ~ 3.803)	0.014
Transverse	35(28.5%)	33(26.8%)	2(1.6%)	0.136 (0.028 ~ 0.663)	0.014
Spiral	49(39.8%)	35(28.5%)	14(11.4%)	0.900(0.359 ~ 2.259)	0.822
Comminuted	39(31.7%)	27(22%)	12(9.8%)	1.0(reference)	0.040
Open reduction					
Open	45(36.6%)	40(32.5%)	5(4.1%)	0.299(0.105 ~ 0.854)	0.024
Close	78(63.4%)	55(44.7%)	23(18.7%)		
Diabetes					
Yes	6(4.9%)	3(2.4%)	3(2.4%)	3.68(0.7 ~ 19.359)	0.124
No	117(95.1%)	92(74.8%)	25(20.3%)		
Smoking					
Yes	35(28.5%)	24(19.5%)	11(8.9%)	1.914(0.787 ~ 4.654)	0.152
No	88(71.5%)	71(57.7%)	17(13.8%)		
High-energy damage					
Yes	85 (69.1%)	68 (55.3%)	17 (13.8%)	0.614(0.255 ~ 1.479)	0.277
No	38 (30.9%)	27 (22%)	11 (8.9%)		
Open fracture					
Open	34(27.6%)	27(22%)	7(5.7%)	0.84(0.32 ~ 2.203)	0.722
Close	89(72.4%)	68(55.3%)	21(17.1%)		
Surgical approach					
Suprapatellar	37(30.1%)	34(27.6%)	3(2.4%)		
Subpatellar	86(69.9%)	61(49.6%)	25(20.3%)	4.645(1.306 ~ 16.521)	0.018
TFI ratio	1.37 ± 0.03	1.3 ± 0.03	1.64 ± 0.06	19.175 (4.884 ~ 75.279)	0.001*

*OR*, odds ratio; *CI*, confidence interval; *TFI ratio*, the ratio of fracture site diameter to tibial isthmus diameter

∗ Significant difference, *p* < 0.05

### Risk factors associated with complications

The results of single-factor logistic regression analysis showed several factors related to the occurrence of complications. Fracture type, surgical method of open reduction, superior patellar approach, and TFI ratio value were significantly correlated with postoperative complications of intramedullary nailing of a tibial fracture (Table 2). In the multivariate logistic regression analysis, the incidence of complications was significantly correlated with open reduction surgery (*P* = 0.002) and the suprapatellar/subpatellar intramedullary nail approach (*P* = 0.002). TFI ratio was identified as an independent risk factor for complications (*P* = 0.001*) (Table 3).

**Table 3:** Multivariate logistic regression analysis of the risk factors associated with complications

Predictor	AOR	95% CI	*P*
Fracture type			
Transverse	0.184	0.031 ~ 1.098	0.063
Spiral	0.605	0.179 ~ 1.197	2.047
Comminuted	1.0	(Reference)	0.177
Open reduction	0.097	0.022 ~ 0.421	0.002
Subpatellar	13.577	2.634 ~ 69.988	0.002
TFI ratio	70.968	8.119 ~ 620.352	0.001*

*AOR*, adjusted odds ratio; *CI*, confidence interval; *TFI ratio*, the ratio of fracture site diameter to tibial isthmus diameter

∗ Significant difference, *p* < 0.05

**Fig. 2:**
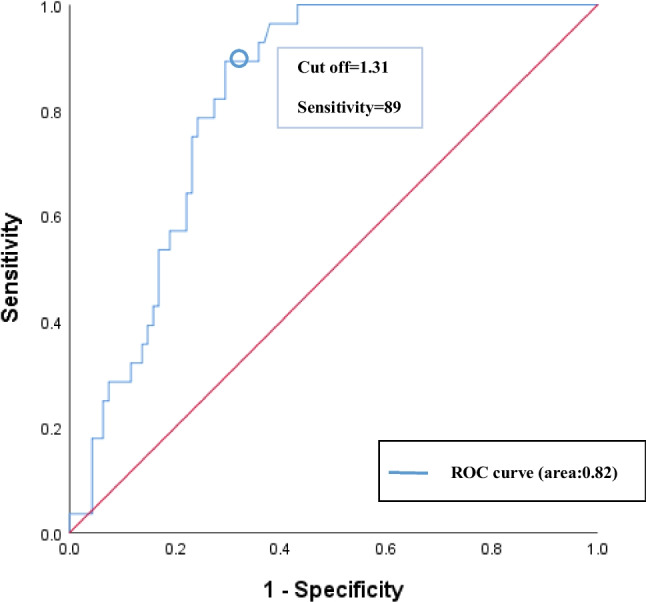
Receiver operating characteristic (ROC) curves were generated to establish a threshold of TFI ratio to predict the occurrence of complications

Using the TFI ratio as a predictor of complications, an ROC curve was generated to establish a threshold. The ROC curve showed that a TFI ratio ≥ 1.31 had a sensitivity of 0.89 and a specificity of 0.71 for predicting complications (Fig. 2). With a TFI ratio of 1.31 as the critical value, patients with TFI ratios < 1.31 (3/70; 4.3%) had significantly lower complication rates than those with TFI ratios ≥ 1.31 (25/53; 47.2%) (*P* = 0.001), and the area under the ROC curve was 0.82 (Fig. 2). In addition, 89.3% (25/28) of patients with complications had TFI ratio ≥ 1.31.

The ROC curve showed that the sensitivity and specificity of a TFI ratio ≥ 1.31 for predicting the occurrence of complications were 89% and 71%, respectively. The AUC (area under the curve) was 0.82.

## Discussion

The TFI ratio was a parameter used to evaluate the stability of the tibial intramedullary nail in combination with the length of the distal fracture and the width of the medullary cavity. The higher the ratio, the wider the pulp cavity at the fracture site and the shorter the effective working length of the intramedullary nail, both of which hinder the stability of the tibial intramedullary nail fixation. Multivariate logistic regression analysis determined that a high TFI ratio was an independent risk factor for postoperative complications.

Although intramedullary nailing is considered the standard treatment for fractures of the tibial shaft, it is also associated with an increased risk of bone nonunion or misalignment [12, 13]. When the intramedullary nail is fixed in the middle and lower tibia, the short working length and large metaphyseal volume make it difficult to achieve rigid cortical contact with the distal end of the fracture [14]. Previous studies have shown that intramedullary nailing of distal tibial fractures often results in varus or valgus malformations, torsion malformations, and nonunion [15–18]. A valgus deformity is the most commonly reported abnormality. While a variety of factors contribute to functional recovery and avoid the risk of post-traumatic arthritis, poor alignment after nailing of tibial shaft fractures has been reported to negatively impact patient outcomes, including excessive subtalar stiffness, reduced exercise tolerance, and reduced patient satisfaction [19–21]. The complication rate of tibial fractures has been reported to be as high as 14 to 58% [22, 23]. The most likely factor determining the occurrence of these complications may be the instability caused by the difference between the diameter of the intramedullary nail and the diameter of the metaphysis. Mugundhan et al. analyzed 20 patients in whom the isthmus level of the medullary canal did not match the fracture site, and the results showed that there was an obvious mismatch resulting in the metaphyseal end not contacting the intramedullary nail [24]. In the absence of contact with the metaphyseal cortex, the intramedullary nail may shift along the interlocking screw, especially if there is a fracture of more than one plane [25].

Kim et al. reported that complications were more likely in subisthmic fractures with shorter working lengths and wider medullary ducts at the distal end [9]. A sufficient working length is helpful for preoperative planning and intraoperative judgment [26]. A long lever arm, short distal locking and a relatively wide metaphysis, greater distal lever force, and automatic fracture alignment due to lack of contact with the cortex and considerable intramedullary nail mobility can lead to higher rates of misalignment and an increased risk of screw failure with locking screws [27]. Our data showed that complications occurred in 28 of 123 patients with middle and lower tibial fractures, with an incidence of 22.4%. Twenty-two patients had malunion, and 6 patients had delayed union.

**Fig. 3:**
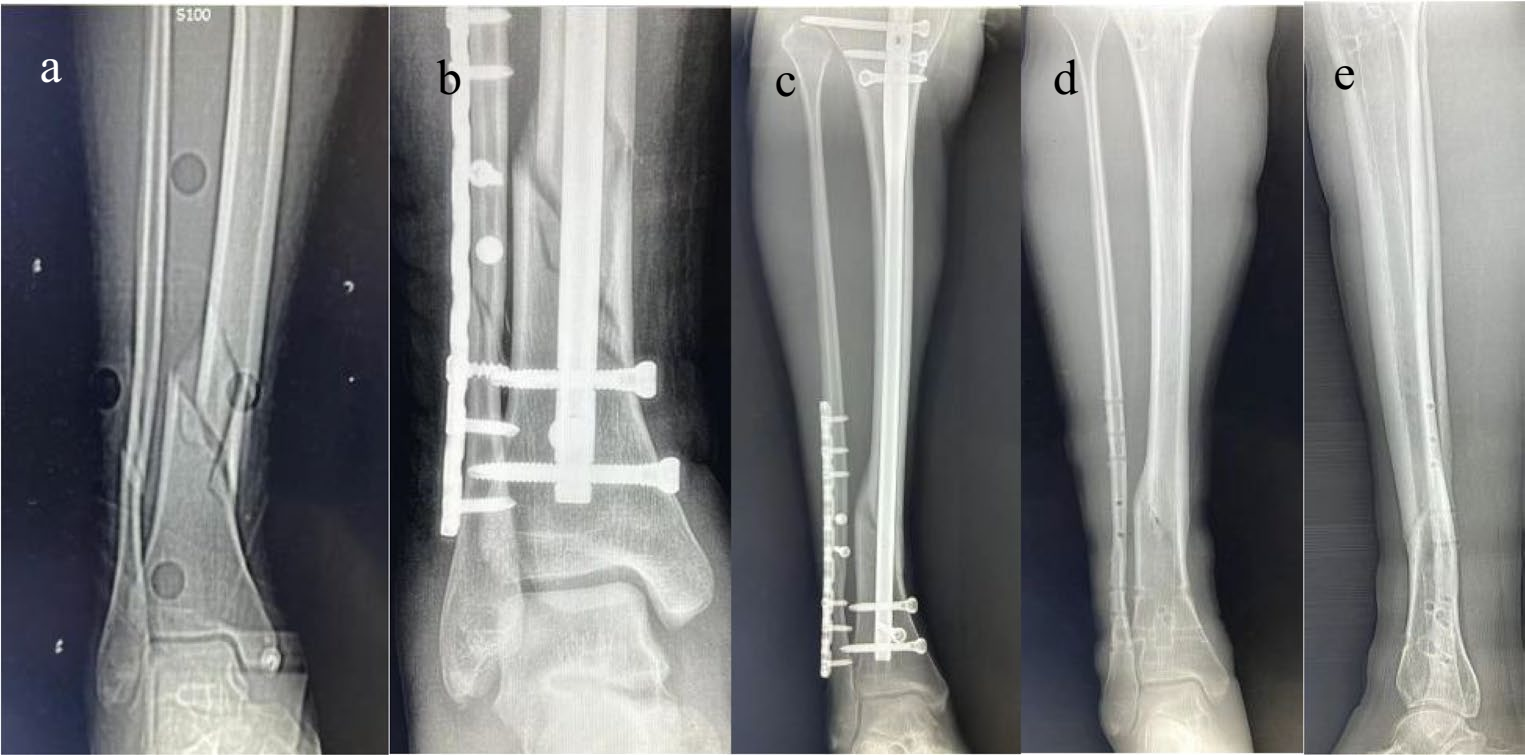
Distal tibial fracture, poor alignment after intramedullary nailing, 1-year follow-up, malunion radiographs. (**a**) Preoperative radiographs with a TFI ratio of 1.99; (**b**) postoperative reexamination of the radiographs showed poor alignment, and the alignment of the anteroposterior radiographs turned inward at an angle of 7°; (**c**) 5 months after surgery, the fracture line appeared slightly blurred; (**d**, **e**) fracture malunion occurred after the internal fixation device was removed

**Fig. 4:**
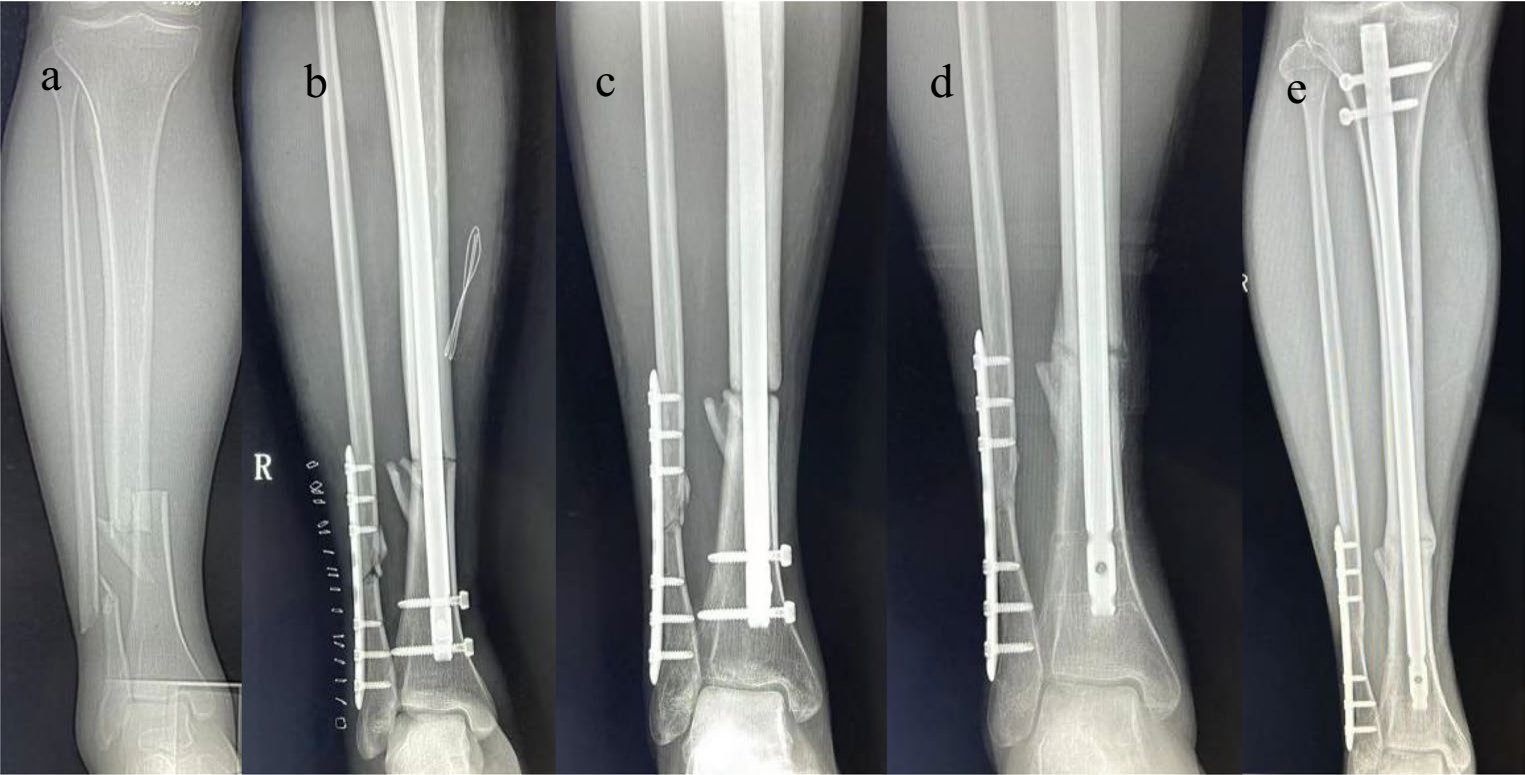
Radiographs showing delayed healing after 1.5 years of follow-up after intramedullary nailing of a distal tibia fracture. (**a**) Preoperative radiographs with a TFI ratio of 1.45; (**b**) postoperative reexamination of radiographs showed poor alignment, and the anteroposterior radiographs were inverted at an angle of 4°; (**c**) 5 months after surgery, bone resorption occurred at the fracture end, an obvious fracture line was observed, and delayed healing occurred; (**d**) 5 months after the distal locking device was removed, reexamination of the radiographs revealed blurred fracture lines and callus junction fractures; (**e**) 1 and a half years after surgery, the fracture line was barely visible, a callus junction fracture occurred, and fracture healing occurred

The most important finding of this study is the TFI ratio as a new parameter to predict the complications of tibial IM nails in patients with middle and lower tibial fractures. A TFI ratio ≥ 1.31 was a strong predictor of postoperative complications (Figs. 3 and 4). Therefore, additional fixation techniques or alternative fixation methods may be considered to improve surgical outcomes in patients with TFI ratio values of ≥ 1.31 that are found preoperatively.

In this study, the complication rate in patients who received closed reduction was higher than that in patients who received open reduction. If the TFI ratio is greater than or equal to 1.31, a closed reduction attempt may result in poor reduction. Limited open reduction of the fracture may be attempted if necessary, and intramedullary nailing may reduce the risk of postoperative complications when satisfactory reduction is achieved [28]. In addition, the subpatellar approach is associated with a higher complication rate than the suprapatellar approach. The suprapatellar approach does not require knee flexion to ensure intramedullary nail insertion and is more effective for fracture reduction, which is an important factor in reducing complications [29].

The distal tibial medullary canal is wider than the isthmus of the shaft, resulting in a higher incidence of inadequate reduction and poor healing. To reduce the risk of poor reduction, loss of reduction, and malunion of distal tibial fractures, several surgical techniques and designs have been used to improve surgical outcomes. The barrier screw helps place the intramedullary nail in the desired position and prevents it from migrating by reducing the metaphyseal space [30]. Multidirectional distal locking using at least two screws has been shown to reduce screw failure and fracture nonunion, thus contributing to the biomechanical stability of distal tibial fractures [31]. Other techniques include the use of a single cortical plate for auxiliary fixation and the use of an eccentric end fixation technique for intramedullary nails [32].

There are some limitations to this study. First, this was a retrospective study, so there was inherent bias. Second, all patients in this study came from a single trauma center, and the number was limited. Therefore, a large multicenter sample study is needed to confirm our findings, and future studies with many patients will provide stronger evidence. Third, rotation and length deformities were not examined in this radiographic study and should be further investigated in the future.

## Conclusion

The results of this study suggest that a wider intramedullary diameter and a shorter fixed length at the fracture site are associated with a higher incidence of complications after tibial intramedullary nailing. The TFI ratio may be used as a reliable parameter for predicting complications after such surgery. For patients with a high TFI ratio (≥ 1.31), additional reduction and fixation techniques may be needed to obtain and maintain fracture reduction (Figs. 3 and 4).

## Data Availability

The datasets generated and/or analyzed during the current study are not publicly available, as they contain information that could compromise the privacy of research participants, but they are available from the corresponding author upon reasonable request.

## Abbreviations

*TFI ratio*: The ratio of tibial fracture site diameter to isthmus diameter
*EWLD*: Distal effective working length
*RUST*: Radiographic union scale for tibial fractures
*OR*: Odds ratio
*AOR*: Adjusted odds ratio
*CI*: Confidence interval
